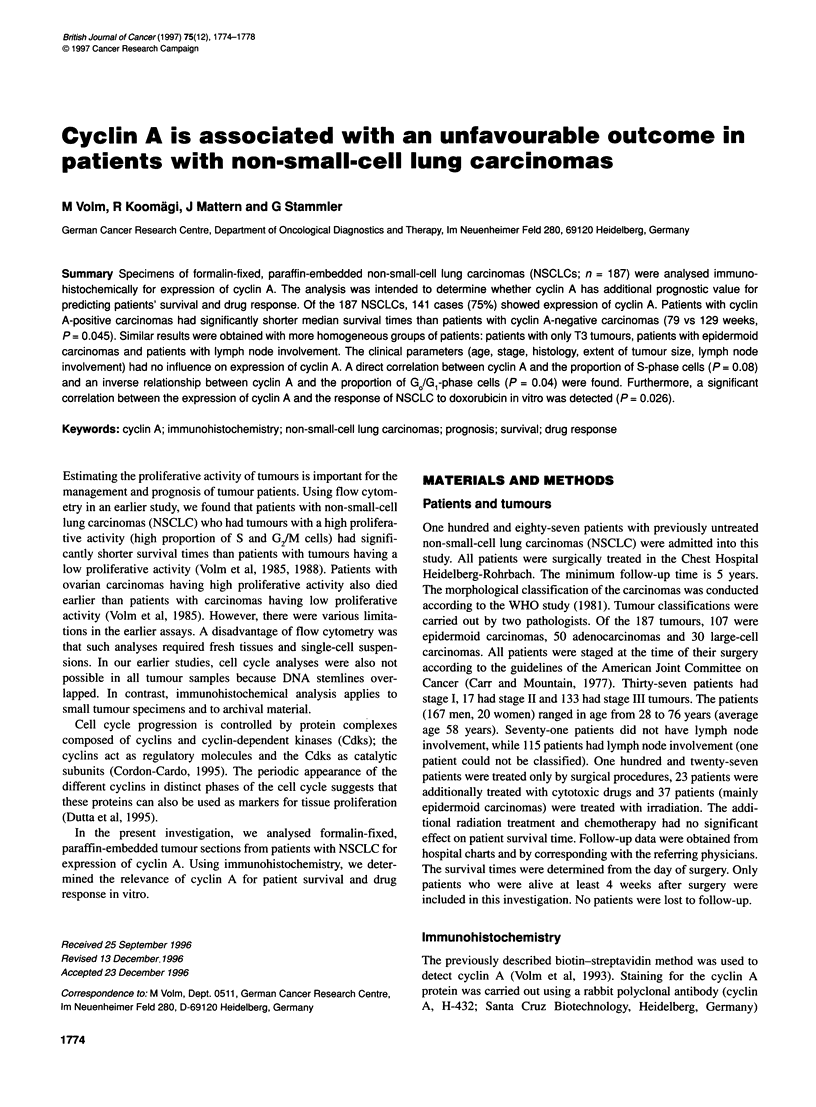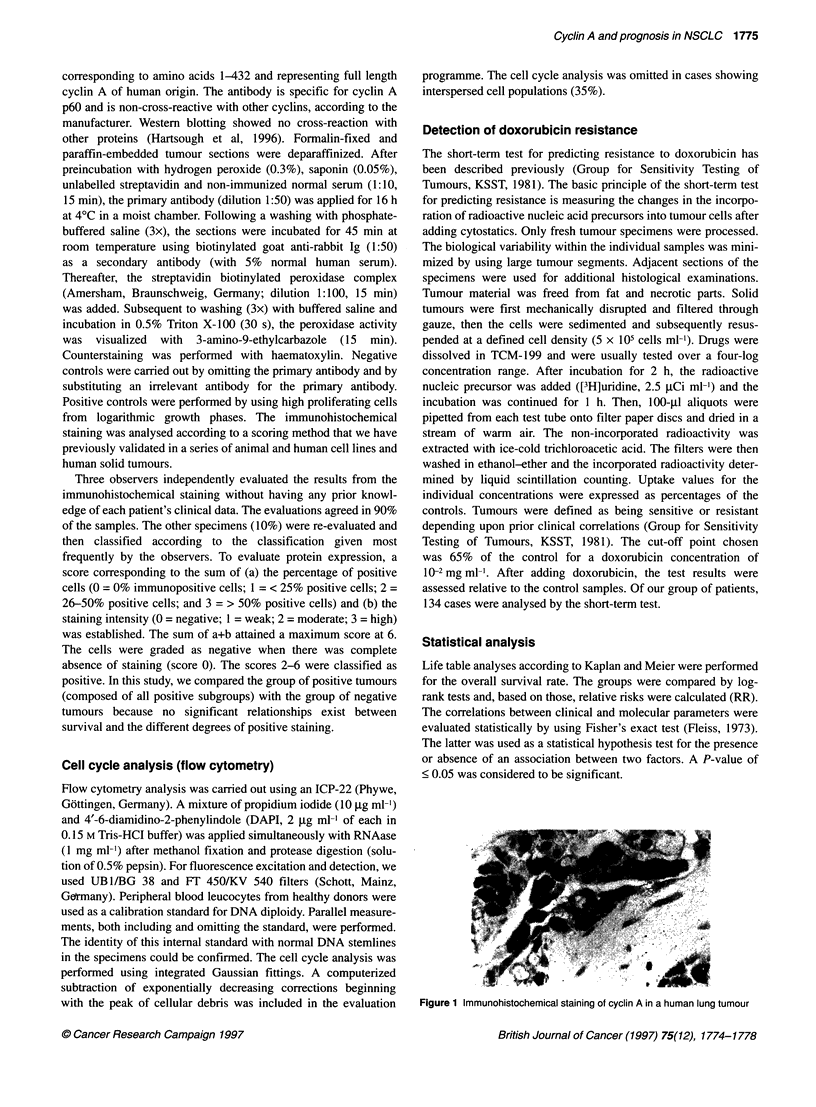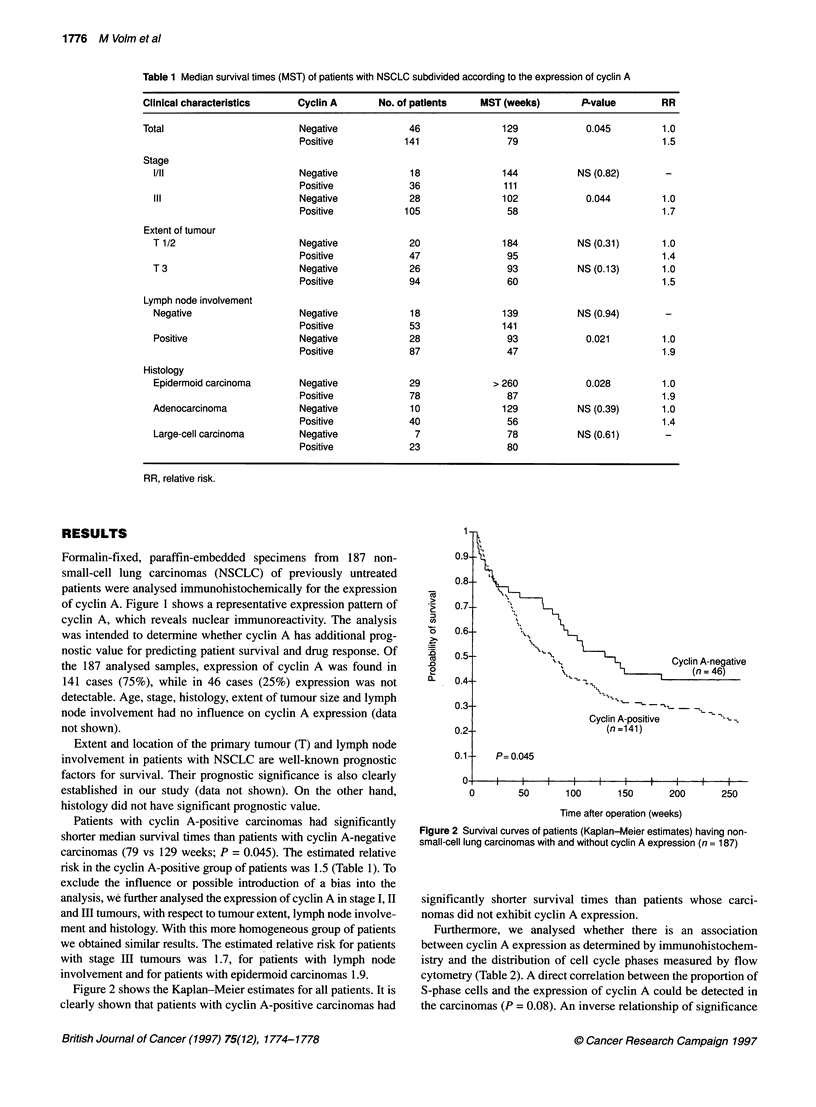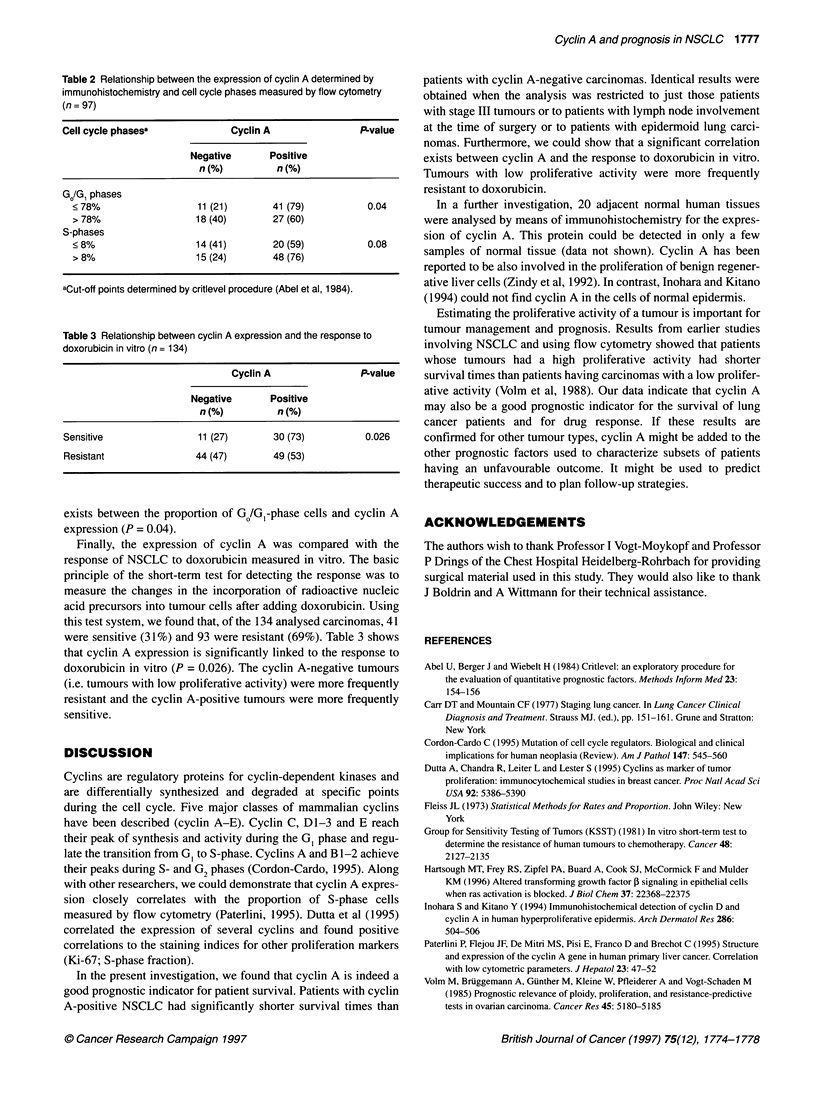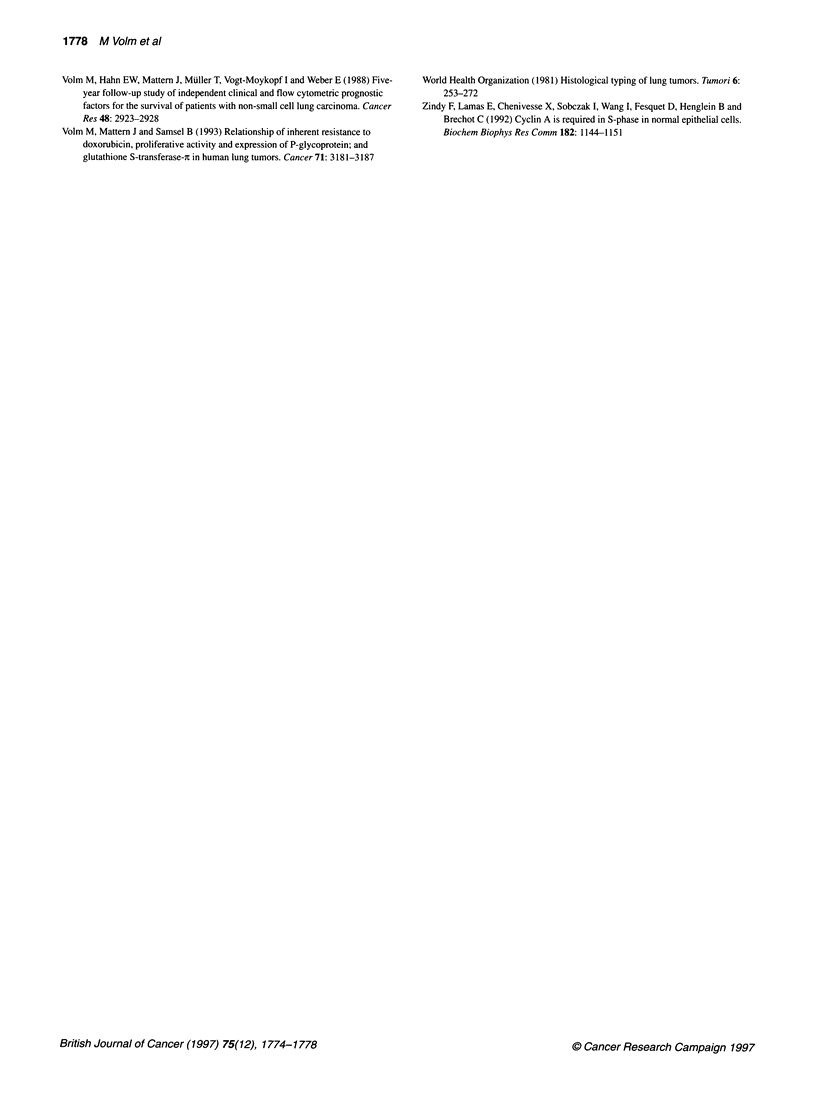# Cyclin A is associated with an unfavourable outcome in patients with non-small-cell lung carcinomas.

**DOI:** 10.1038/bjc.1997.302

**Published:** 1997

**Authors:** M. Volm, R. KoomÃ¤gi, J. Mattern, G. Stammler

**Affiliations:** German Cancer Research Centre, Department of Oncological Diagnostics and Therapy, Heidelberg.

## Abstract

**Images:**


British Journal of Cancer (1997) 75(12), 1774–1778
© 1997 Cancer Research Campaign

### Cyclin A is associated with an unfavourable outcome in patients with non-small-cell lung carcinomas

**M Volm, R Koomägi, J Mattern and G Stammler**

German Cancer Research Centre, Department of Oncological Diagnostics and Therapy, Im Neuenheimer Feld 280, 69120 Heidelberg, Germany

**Summary** Specimens of formalin-fixed, paraffin-embedded non-small-cell lung carcinomas (NSCLCs; $n$ = 187) were analysed immunohistochemically for expression of cyclin A. The analysis was intended to determine whether cyclin A has additional prognostic value for predicting patients' survival and drug response. Of the 187 NSCLCs, 141 cases (75%) showed expression of cyclin A. Patients with cyclin A-positive carcinomas had significantly shorter median survival times than patients with cyclin A-negative carcinomas (79 vs 129 weeks, $P = 0.045$). Similar results were obtained with more homogeneous groups of patients: patients with only T3 tumours, patients with epidermoid carcinomas and patients with lymph node involvement. The clinical parameters (age, stage, histology, extent of tumour size, lymph node involvement) had no influence on expression of cyclin A. A direct correlation between cyclin A and the proportion of S-phase cells ($P = 0.08$) and an inverse relationship between cyclin A and the proportion of $G_0/G_1$-phase cells ($P = 0.04$) were found. Furthermore, a significant correlation between the expression of cyclin A and the response of NSCLC to doxorubicin in vitro was detected ($P = 0.026$).

**Keywords:** cyclin A; immunohistochemistry; non-small-cell lung carcinomas; prognosis; survival; drug response

Estimating the proliferative activity of tumours is important for the management and prognosis of tumour patients. Using flow cytometry in an earlier study, we found that patients with non-small-cell lung carcinomas (NSCLC) who had tumours with a high proliferative activity (high proportion of S and $G_2$/M cells) had significantly shorter survival times than patients with tumours having a low proliferative activity (Volm et al, 1985, 1988). Patients with ovarian carcinomas having high proliferative activity also died earlier than patients with carcinomas having low proliferative activity (Volm et al, 1985). However, there were various limitations in the earlier assays. A disadvantage of flow cytometry was that such analyses required fresh tissues and single-cell suspensions. In our earlier studies, cell cycle analyses were also not possible in all tumour samples because DNA stemlines overlapped. In contrast, immunohistochemical analysis applies to small tumour specimens and to archival material.

Cell cycle progression is controlled by protein complexes composed of cyclins and cyclin-dependent kinases (Cdks); the cyclins act as regulatory molecules and the Cdks as catalytic subunits (Cordon-Cardo, 1995). The periodic appearance of the different cyclins in distinct phases of the cell cycle suggests that these proteins can also be used as markers for tissue proliferation (Dutta et al, 1995).

In the present investigation, we analysed formalin-fixed, paraffin-embedded tumour sections from patients with NSCLC for expression of cyclin A. Using immunohistochemistry, we determined the relevance of cyclin A for patient survival and drug response in vitro.

*Received 25 September 1996*
*Revised 13 December 1996*
*Accepted 23 December 1996*

*Correspondence to:* M Volm, Dept. 0511, German Cancer Research Centre, Im Neuenheimer Feld 280, D-69120 Heidelberg, Germany

### MATERIALS AND METHODS

### Patients and tumours

One hundred and eighty-seven patients with previously untreated non-small-cell lung carcinomas (NSCLC) were admitted into this study. All patients were surgically treated in the Chest Hospital Heidelberg-Rohrbach. The minimum follow-up time is 5 years. The morphological classification of the carcinomas was conducted according to the WHO study (1981). Tumour classifications were carried out by two pathologists. Of the 187 tumours, 107 were epidermoid carcinomas, 50 adenocarcinomas and 30 large-cell carcinomas. All patients were staged at the time of their surgery according to the guidelines of the American Joint Committee on Cancer (Carr and Mountain, 1977). Thirty-seven patients had stage I, 17 had stage II and 133 had stage III tumours. The patients (167 men, 20 women) ranged in age from 28 to 76 years (average age 58 years). Seventy-one patients did not have lymph node involvement, while 115 patients had lymph node involvement (one patient could not be classified). One hundred and twenty-seven patients were treated only by surgical procedures, 23 patients were additionally treated with cytotoxic drugs and 37 patients (mainly epidermoid carcinomas) were treated with irradiation. The additional radiation treatment and chemotherapy had no significant effect on patient survival time. Follow-up data were obtained from hospital charts and by corresponding with the referring physicians. The survival times were determined from the day of surgery. Only patients who were alive at least 4 weeks after surgery were included in this investigation. No patients were lost to follow-up.

### Immunohistochemistry

The previously described biotin–streptavidin method was used to detect cyclin A (Volm et al, 1993). Staining for the cyclin A protein was carried out using a rabbit polyclonal antibody (cyclin A, H-432; Santa Cruz Biotechnology, Heidelberg, Germany)

corresponding to amino acids 1–432 and representing full length cyclin A of human origin. The antibody is specific for cyclin A p60 and is non-cross-reactive with other cyclins, according to the manufacturer. Western blotting showed no cross-reaction with other proteins (Hartsough et al, 1996). Formalin-fixed and paraffin-embedded tumour sections were deparaffinized. After preincubation with hydrogen peroxide (0.3%), saponin (0.05%), unlabelled streptavidin and non-immunized normal serum (1:10, 15 min), the primary antibody (dilution 1:50) was applied for 16 h at 4°C in a moist chamber. Following a washing with phosphate-buffered saline (3×), the sections were incubated for 45 min at room temperature using biotinylated goat anti-rabbit Ig (1:50) as a secondary antibody (with 5% normal human serum). Thereafter, the streptavidin biotinylated peroxidase complex (Amersham, Braunschweig, Germany; dilution 1:100, 15 min) was added. Subsequent to washing (3×) with buffered saline and incubation in 0.5% Triton X-100 (30 s), the peroxidase activity was visualized with 3-amino-9-ethylcarbazole (15 min). Counterstaining was performed with haematoxylin. Negative controls were carried out by omitting the primary antibody and by substituting an irrelevant antibody for the primary antibody. Positive controls were performed by using high proliferating cells from logarithmic growth phases. The immunohistochemical staining was analysed according to a scoring method that we have previously validated in a series of animal and human cell lines and human solid tumours.

Three observers independently evaluated the results from the immunohistochemical staining without having any prior knowledge of each patient's clinical data. The evaluations agreed in 90% of the samples. The other specimens (10%) were re-evaluated and then classified according to the classification given most frequently by the observers. To evaluate protein expression, a score corresponding to the sum of (a) the percentage of positive cells (0 = 0% immunopositive cells; 1 = < 25% positive cells; 2 = 26–50% positive cells; and 3 = > 50% positive cells) and (b) the staining intensity (0 = negative; 1 = weak; 2 = moderate; 3 = high) was established. The sum of a+b attained a maximum score at 6. The cells were graded as negative when there was complete absence of staining (score 0). The scores 2–6 were classified as positive. In this study, we compared the group of positive tumours (composed of all positive subgroups) with the group of negative tumours because no significant relationships exist between survival and the different degrees of positive staining.

### Cell cycle analysis (flow cytometry)

Flow cytometry analysis was carried out using an ICP-22 (Phywe, Göttingen, Germany). A mixture of propidium iodide (10 μg $ml^{-1}$) and 4′-6-diamidino-2-phenylindole (DAPI, 2 μg $ml^{-1}$ of each in 0.15 M Tris-HCI buffer) was applied simultaneously with RNAase (1 mg $ml^{-1}$) after methanol fixation and protease digestion (solution of 0.5% pepsin). For fluorescence excitation and detection, we used UB1/BG 38 and FT 450/KV 540 filters (Schott, Mainz, Germany). Peripheral blood leucocytes from healthy donors were used as a calibration standard for DNA diploidy. Parallel measurements, both including and omitting the standard, were performed. The identity of this internal standard with normal DNA stemlines in the specimens could be confirmed. The cell cycle analysis was performed using integrated Gaussian fittings. A computerized subtraction of exponentially decreasing corrections beginning with the peak of cellular debris was included in the evaluation programme. The cell cycle analysis was omitted in cases showing interspersed cell populations (35%).

### Detection of doxorubicin resistance

The short-term test for predicting resistance to doxorubicin has been described previously (Group for Sensitivity Testing of Tumours, KSST, 1981). The basic principle of the short-term test for predicting resistance is measuring the changes in the incorporation of radioactive nucleic acid precursors into tumour cells after adding cytostatics. Only fresh tumour specimens were processed. The biological variability within the individual samples was minimized by using large tumour segments. Adjacent sections of the specimens were used for additional histological examinations. Tumour material was freed from fat and necrotic parts. Solid tumours were first mechanically disrupted and filtered through gauze, then the cells were sedimented and subsequently resuspended at a defined cell density ($5 \times 10^5$ cells $ml^{-1}$). Drugs were dissolved in TCM-199 and were usually tested over a four-log concentration range. After incubation for 2 h, the radioactive nucleic precursor was added ([$^3$H]uridine, 2.5 μCi $ml^{-1}$) and the incubation was continued for 1 h. Then, 100-μl aliquots were pipetted from each test tube onto filter paper discs and dried in a stream of warm air. The non-incorporated radioactivity was extracted with ice-cold trichloroacetic acid. The filters were then washed in ethanol–ether and the incorporated radioactivity determined by liquid scintillation counting. Uptake values for the individual concentrations were expressed as percentages of the controls. Tumours were defined as being sensitive or resistant depending upon prior clinical correlations (Group for Sensitivity Testing of Tumours, KSST, 1981). The cut-off point chosen was 65% of the control for a doxorubicin concentration of $10^{-2}$ mg $ml^{-1}$. After adding doxorubicin, the test results were assessed relative to the control samples. Of our group of patients, 134 cases were analysed by the short-term test.

### Statistical analysis

Life table analyses according to Kaplan and Meier were performed for the overall survival rate. The groups were compared by log-rank tests and, based on those, relative risks were calculated (RR). The correlations between clinical and molecular parameters were evaluated statistically by using Fisher's exact test (Fleiss, 1973). The latter was used as a statistical hypothesis test for the presence or absence of an association between two factors. A $P$-value of $\leq 0.05$ was considered to be significant.

**Figure 1** Immunohistochemical staining of cyclin A in a human lung tumour

© Cancer Research Campaign 1997

**Table 1** Median survival times (MST) of patients with NSCLC subdivided according to the expression of cyclin A

| Clinical characteristics | Cyclin A | No. of patients | MST (weeks) | *P*-value | RR |
|---|---|---|---|---|---|
| Total | Negative | 46 | 129 | 0.045 | 1.0 |
| | Positive | 141 | 79 | | 1.5 |
| Stage | | | | | |
| I/II | Negative | 18 | 144 | NS (0.82) | – |
| | Positive | 36 | 111 | | |
| III | Negative | 28 | 102 | 0.044 | 1.0 |
| | Positive | 105 | 58 | | 1.7 |
| Extent of tumour | | | | | |
| T 1/2 | Negative | 20 | 184 | NS (0.31) | 1.0 |
| | Positive | 47 | 95 | | 1.4 |
| T 3 | Negative | 26 | 93 | NS (0.13) | 1.0 |
| | Positive | 94 | 60 | | 1.5 |
| Lymph node involvement | | | | | |
| Negative | Negative | 18 | 139 | NS (0.94) | – |
| | Positive | 53 | 141 | | |
| Positive | Negative | 28 | 93 | 0.021 | 1.0 |
| | Positive | 87 | 47 | | 1.9 |
| Histology | | | | | |
| Epidermoid carcinoma | Negative | 29 | > 260 | 0.028 | 1.0 |
| | Positive | 78 | 87 | | 1.9 |
| Adenocarcinoma | Negative | 10 | 129 | NS (0.39) | 1.0 |
| | Positive | 40 | 56 | | 1.4 |
| Large-cell carcinoma | Negative | 7 | 78 | NS (0.61) | – |
| | Positive | 23 | 80 | | |

RR, relative risk.

### RESULTS

Formalin-fixed, paraffin-embedded specimens from 187 non-small-cell lung carcinomas (NSCLC) of previously untreated patients were analysed immunohistochemically for the expression of cyclin A. Figure 1 shows a representative expression pattern of cyclin A, which reveals nuclear immunoreactivity. The analysis was intended to determine whether cyclin A has additional prognostic value for predicting patient survival and drug response. Of the 187 analysed samples, expression of cyclin A was found in 141 cases (75%), while in 46 cases (25%) expression was not detectable. Age, stage, histology, extent of tumour size and lymph node involvement had no influence on cyclin A expression (data not shown).

Extent and location of the primary tumour (T) and lymph node involvement in patients with NSCLC are well-known prognostic factors for survival. Their prognostic significance is also clearly established in our study (data not shown). On the other hand, histology did not have significant prognostic value.

Patients with cyclin A-positive carcinomas had significantly shorter median survival times than patients with cyclin A-negative carcinomas (79 vs 129 weeks; $P = 0.045$). The estimated relative risk in the cyclin A-positive group of patients was 1.5 (Table 1). To exclude the influence or possible introduction of a bias into the analysis, we further analysed the expression of cyclin A in stage I, II and III tumours, with respect to tumour extent, lymph node involvement and histology. With this more homogeneous group of patients we obtained similar results. The estimated relative risk for patients with stage III tumours was 1.7, for patients with lymph node involvement and for patients with epidermoid carcinomas 1.9.

Figure 2 shows the Kaplan–Meier estimates for all patients. It is clearly shown that patients with cyclin A-positive carcinomas had

**Figure 2** Survival curves of patients (Kaplan–Meier estimates) having non-small-cell lung carcinomas with and without cyclin A expression ($n = 187$)

significantly shorter survival times than patients whose carcinomas did not exhibit cyclin A expression.

Furthermore, we analysed whether there is an association between cyclin A expression as determined by immunohistochemistry and the distribution of cell cycle phases measured by flow cytometry (Table 2). A direct correlation between the proportion of S-phase cells and the expression of cyclin A could be detected in the carcinomas ($P = 0.08$). An inverse relationship of significance

© Cancer Research Campaign 1997

**Table 2** Relationship between the expression of cyclin A determined by immunohistochemistry and cell cycle phases measured by flow cytometry ($n$ = 97)

| Cell cycle phases[a] | Cyclin A | | P-value |
|---|---|---|---|
| | Negative $n$ (%) | Positive $n$ (%) | |
| $G_0/G_1$ phases | | | |
| ≤ 78% | 11 (21) | 41 (79) | 0.04 |
| > 78% | 18 (40) | 27 (60) | |
| S-phases | | | |
| ≤ 8% | 14 (41) | 20 (59) | 0.08 |
| > 8% | 15 (24) | 48 (76) | |

[a]Cut-off points determined by critlevel procedure (Abel et al, 1984).

**Table 3** Relationship between cyclin A expression and the response to doxorubicin in vitro ($n$ = 134)

| | Cyclin A | | P-value |
|---|---|---|---|
| | Negative $n$ (%) | Positive $n$ (%) | |
| Sensitive | 11 (27) | 30 (73) | 0.026 |
| Resistant | 44 (47) | 49 (53) | |

exists between the proportion of $G_0/G_1$-phase cells and cyclin A expression ($P$ = 0.04).

Finally, the expression of cyclin A was compared with the response of NSCLC to doxorubicin measured in vitro. The basic principle of the short-term test for detecting the response was to measure the changes in the incorporation of radioactive nucleic acid precursors into tumour cells after adding doxorubicin. Using this test system, we found that, of the 134 analysed carcinomas, 41 were sensitive (31%) and 93 were resistant (69%). Table 3 shows that cyclin A expression is significantly linked to the response to doxorubicin in vitro ($P$ = 0.026). The cyclin A-negative tumours (i.e. tumours with low proliferative activity) were more frequently resistant and the cyclin A-positive tumours were more frequently sensitive.

### DISCUSSION

Cyclins are regulatory proteins for cyclin-dependent kinases and are differentially synthesized and degraded at specific points during the cell cycle. Five major classes of mammalian cyclins have been described (cyclin A–E). Cyclin C, D1–3 and E reach their peak of synthesis and activity during the $G_1$ phase and regulate the transition from $G_1$ to S-phase. Cyclins A and B1–2 achieve their peaks during S- and $G_2$ phases (Cordon-Cardo, 1995). Along with other researchers, we could demonstrate that cyclin A expression closely correlates with the proportion of S-phase cells measured by flow cytometry (Paterlini, 1995). Dutta et al (1995) correlated the expression of several cyclins and found positive correlations to the staining indices for other proliferation markers (Ki-67; S-phase fraction).

In the present investigation, we found that cyclin A is indeed a good prognostic indicator for patient survival. Patients with cyclin A-positive NSCLC had significantly shorter survival times than patients with cyclin A-negative carcinomas. Identical results were obtained when the analysis was restricted to just those patients with stage III tumours or to patients with lymph node involvement at the time of surgery or to patients with epidermoid lung carcinomas. Furthermore, we could show that a significant correlation exists between cyclin A and the response to doxorubicin in vitro. Tumours with low proliferative activity were more frequently resistant to doxorubicin.

In a further investigation, 20 adjacent normal human tissues were analysed by means of immunohistochemistry for the expression of cyclin A. This protein could be detected in only a few samples of normal tissue (data not shown). Cyclin A has been reported to be also involved in the proliferation of benign regenerative liver cells (Zindy et al, 1992). In contrast, Inohara and Kitano (1994) could not find cyclin A in the cells of normal epidermis.

Estimating the proliferative activity of a tumour is important for tumour management and prognosis. Results from earlier studies involving NSCLC and using flow cytometry showed that patients whose tumours had a high proliferative activity had shorter survival times than patients having carcinomas with a low proliferative activity (Volm et al, 1988). Our data indicate that cyclin A may also be a good prognostic indicator for the survival of lung cancer patients and for drug response. If these results are confirmed for other tumour types, cyclin A might be added to the other prognostic factors used to characterize subsets of patients having an unfavourable outcome. It might be used to predict therapeutic success and to plan follow-up strategies.

### ACKNOWLEDGEMENTS

The authors wish to thank Professor I Vogt-Moykopf and Professor P Drings of the Chest Hospital Heidelberg-Rohrbach for providing surgical material used in this study. They would also like to thank J Boldrin and A Wittmann for their technical assistance.

### REFERENCES

Abel U, Berger J and Wiebelt H (1984) Critlevel: an exploratory procedure for the evaluation of quantitative prognostic factors. *Methods Inform Med* **23**: 154–156

Carr DT and Mountain CF (1977) Staging lung cancer. In *Lung Cancer Clinical Diagnosis and Treatment*. Strauss MJ. (ed.), pp. 151–161. Grune and Stratton: New York

Cordon-Cardo C (1995) Mutation of cell cycle regulators. Biological and clinical implications for human neoplasia (Review). *Am J Pathol* **147**: 545–560

Dutta A, Chandra R, Leiter L and Lester S (1995) Cyclins as marker of tumor proliferation: immunocytochemical studies in breast cancer. *Proc Natl Acad Sci USA* **92**: 5386–5390

Fleiss JL (1973) *Statistical Methods for Rates and Proportion*. John Wiley: New York

Group for Sensitivity Testing of Tumors (KSST) (1981) In vitro short-term test to determine the resistance of human tumours to chemotherapy. *Cancer* **48**: 2127–2135

Hartsough MT, Frey RS, Zipfel PA, Buard A, Cook SJ, McCormick F and Mulder KM (1996) Altered transforming growth factor β signaling in epithelial cells when ras activation is blocked. *J Biol Chem* **37**: 22368–22375

Inohara S and Kitano Y (1994) Immunohistochemical detection of cyclin D and cyclin A in human hyperproliferative epidermis. *Arch Dermatol Res* **286**: 504–506

Paterlini P, Flejou JF, De Mitri MS, Pisi E, Franco D and Brechot C (1995) Structure and expression of the cyclin A gene in human primary liver cancer. Correlation with low cytometric parameters. *J Hepatol* **23**: 47–52

Volm M, Brüggemann A, Günther M, Kleine W, Pfleiderer A and Vogt-Schaden M (1985) Prognostic relevance of ploidy, proliferation, and resistance-predictive tests in ovarian carcinoma. *Cancer Res* **45**: 5180–5185

© Cancer Research Campaign 1997

Volm M, Hahn EW, Mattern J, Müller T, Vogt-Moykopf I and Weber E (1988) Five-year follow-up study of independent clinical and flow cytometric prognostic factors for the survival of patients with non-small cell lung carcinoma. *Cancer Res* **48**: 2923–2928

Volm M, Mattern J and Samsel B (1993) Relationship of inherent resistance to doxorubicin, proliferative activity and expression of P-glycoprotein; and glutathione S-transferase-π in human lung tumors. *Cancer* **71**: 3181–3187

World Health Organization (1981) Histological typing of lung tumors. *Tumori* **6**: 253–272

Zindy F, Lamas E, Chenivesse X, Sobczak I, Wang I, Fesquet D, Henglein B and Brechot C (1992) Cyclin A is required in S-phase in normal epithelial cells. *Biochem Biophys Res Comm* **182**: 1144–1151

© Cancer Research Campaign 1997